# Systematic Knowledge Management of Construction Safety Standards Based on Knowledge Graphs: A Case Study in China

**DOI:** 10.3390/ijerph182010692

**Published:** 2021-10-12

**Authors:** Yukun Jiang, Xin Gao, Wenxin Su, Jinrong Li

**Affiliations:** School of Economics and Management, Tongji University, Shanghai 200092, China; 1930324@tongji.edu.cn (Y.J.); 1930325@tongji.edu.cn (W.S.); 1932758@tongji.edu.cn (J.L.)

**Keywords:** construction safety standards, knowledge graph, domain knowledge, knowledge management, standards planning

## Abstract

Construction safety standards (CSS) have knowledge characteristics, but few studies have introduced knowledge graphs (KG) as a tool into CSS management. In order to improve CSS knowledge management, this paper first analyzed the knowledge structure of 218 standards and obtained three knowledge levels of CSS. Second, a concept layer was designed which consisted of five levels of concepts and eight types of relationships. Third, an entity layer containing 147 entities was constructed via entity identification, attribute extraction and entity extraction. Finally, 177 nodes and 11 types of attributes were collected and the construction of a knowledge graph of construction safety standard (KGCSS) was completed using knowledge storage. Furthermore, we implemented knowledge inference and obtained CSS planning, i.e., the list of standard work plans used to guide the development and revision of CSS. In addition, we conducted CSS knowledge retrieval; a process which supports interrogative input. The construction of KGCSS thus facilitates the analysis, querying, and sharing of safety standards knowledge.

## 1. Introduction

Construction is a risky industry [1,2]. Construction workers spend most of their time on site [3], and dynamic and complex site operations expose them to many unsafe factors [4,5]. According to previous studies, 22% of occupational fatalities in the United States, 27.2% in the United Kingdom, and 27.6% in South Korea occurred in the construction industry [6,7,8]. Extensive statistics show that the risk of fatal construction accidents is five times greater than in other industries, and the casualty rate is three times higher [9,10,11]. It has been shown that the violation of safety standards in high-risk industries is a significant cause of accidents [12], indicating that safety compliance is critical to maintaining occupational safety. In addition, the reason why workers are violating safety standards needs to be explained so that safety compliance can be improved.

Construction safety standards (CSSs) refer to the common use and reuse of normative documents that were developed by consensus and approved by a recognized authority in order to obtain the optimum degree of construction safety [13]. In practice, safety cannot be achieved without the implementation of a standard of behavior among contractors [14]. Therefore, safety standards are considered to be an important component of safety control. They are utilized to regulate the behavior of all parties engaged in a construction project. Effective management and implementation of standards are conducive to safety improvement [15].

The current state of CSS application has exposed a series of problems in standard management. In China, for example, the number of CSS is large and the integration is poor. Due to the lack of a unified approach to CSS [16], a significant amount of development in construction and science and technology in China has led to the proliferation of various standards [17]. According to incomplete statistics, there are more than 60 national CSSs, more than 40 industry standards, more than 90 local standards, and more than 10 enterprise standards. The large number of standards results in duplications, inconvenience of use, and a waste of compilation resources. Because contractors make comparisons and filtrations in the application of standards, the number of CSS with a high-frequency of use is less than 20. Second, there are contradictions between individual CSSs, leading to difficulties in governmental supervision and contractors’ production. For example, “Wire Rope Grips” stipulates that if a wire rope’s diameter is not greater than 9 mm, the rope card shall not be less than three groups. However, it is required that a rope card shall not be less than three groups if a wire rope’s diameter is not greater than 18 mm in “Steel wire ropes: Safety, Use and maintenance”, “Technical Specification for Safety Operation of Constructional Machinery”, and “Technical Specification for Safety Installation Operation and Dismantlement of Tower Crane in Construction”. Third, standards that are urgently needed in the market are missing, such as “Aluminum Formwork Safety Operation”. In summary, these problems diminish the function of CSS in safety control.

It is critical to address issues in CSS implementation. However, related research is far from complete. Although some research has proposed the implementation of standard management system reform in order to satisfy the objectives of economic and social development [18], this research generally concerns theoretical analysis, rather than concrete feasible measures or the domain of construction safety. Meanwhile, some studies have proposed integrating ISO 9001 and OHSAS 18001 in order to streamline management processes [19], and others have conducted standard knowledge transformation for contractors’ use [20]. Nonetheless, these studies are limited in their capacity to optimize the use of individual standards and are hardly generalizable to the standard management requirements of the construction industry as a whole.

It has been noted that security standards are a form of explicit knowledge. The term “knowledge” differs from “information” in that knowledge consists of facts, skills, and rules collected by individuals over a period [21]. Knowledge is classified into two categories: explicit knowledge and tacit knowledge [22]. Explicit knowledge is formal and can be easily encoded and transferred between individuals in documented and organized forms, such as databases, records, and regulations [21,23]. Safety standards belong to explicit knowledge, and a knowledge management approach can be applied for safety standard management.

From the perspective of knowledge management, the general approach is Semantic Web and knowledge graph (KG), which is extended from Semantic Web. Semantic Web is a graph-based data structure for storing knowledge [24]. KG is an emerging technology for massive knowledge management and intelligent services in the Big Data era [25,26]. Technically speaking, KG is a graph-based knowledge representation and organization method. It uses a set of “subject-predicate-object” triples to represent entities and their relationships [27]. KG can be seen as a huge network where nodes represent domain entities and contacts are regarded as semantic relationships between entities. KG can organize knowledge, establish connections, and solve the problem of “knowledge islands”. The term “knowledge islands” refers to isolated knowledge that lacks connections, and is difficult in terms of enabling knowledge sharing. In addition, KG can realize the functions of knowledge visualization, querying and reasoning [28].

In view of this, KG can establish connections between CSS and resolve issues of poor integration and contradiction. Moreover, KG can perform knowledge visualization, querying and inference, which provides practical measures for standard query and compilation. Therefore, it is feasible to integrate CSS by using KG.

However, few studies have introduced KG into CSS management. To bridge the research gap, KG is adopted to manage CSS knowledge. This paper first analyzes the structure of CSS knowledge, constructs a knowledge graph of construction safety standards (KGCSS), and realizes knowledge query and reasoning. Preliminary research of systematic CSS knowledge management is completed in this paper. The results of this paper not only solve the key problems of standard knowledge management, but also extend the results to other countries and industries.

## 2. Literature Review

### 2.1. CSS Management

The research on CSS management can be divided into two main areas. Firstly, the approach of integrating management of standards has been proposed. Some scholars have attempted to integrate ISO 9001 and OHSAS 18001 to optimize the process, avoid work duplication, and reduce resource input [19]. Secondly, studies have been carried out for the management of specific standards. The technical contents of “Code of Construction for Masonry Structure Engineering” have been analyzed. The influence of masonry mortar use time, the relative moisture content of blocks, and post-tensioning reinforcement on the quality and safety of projects has been derived, which facilitates the ease of comprehension for contractors [29].

As in the related research listed above, there is still a lack of a universal and operational integrated management methodology and tools in the field of CSS. It has been demonstrated that the essence of CSS is a technical system [18] and that knowledge characteristics are part of the technical system framework [30,31]. Previous studies have identified knowledge barriers to CSS application, which refer to the lack of standardization talents and low quality of standard operators [13]. On the other hand, strengthening technical standards knowledge management can reduce users’ time for selecting standard documents [32] and improve project performance. Therefore, it is necessary to organize and manage CSS knowledge in order to improve the sharing and reuse of CSS knowledge.

### 2.2. Knowledge Graph (KG)

KG technology originated from semantic networks in the 1960s [24]. In the 1990s, the idea of “ontology” was introduced into knowledge representation methods [33]. KG was first officially presented by Google in 2012. It refers to a graph-based method for representing and organizing knowledge. KG can associate a large amount of knowledge and information through an organization form and has knowledge reasoning ability. In recent years, the knowledge graph developed from the semantic web has attracted the attention of more and more researchers [34,35,36,37].

General knowledge graphs and domain knowledge graphs are two types of KG [38,39]. General knowledge graphs contain a wide range of general knowledge, and are also called open domain knowledge graphs. WordNet is the result of work to develop a dictionary database, in use since 1985 [40,41]. It implements the adding of simple facts to the knowledge network, but the division of concepts or entities is not very clear. Therefore, Cyc [42] and ConceptNet [43,44] were constructed. They relate concepts to common sense and incorporate complex relationships that exist in the real world. Currently, the most widely used are encyclopedic knowledge graphs, including DBpedia [45], YAGO [46], Freebase [47], Wikidata [48], and others. Wikimedia launched Wikidata in October 2012. Data in Wikidata are basically described by property-value pairs [38]. YAGO is a large semantic knowledge base conducted by the Max Planck Institute in Germany, with one million entities and more than five million facts. Freebase was launched in 2005. It was constructed based on Wikipedia and the idea of swarm intelligence. Encyclopedic knowledge graphs contain a large amount of semi-structured and unstructured data and occupy a central position in open-linked large-scale knowledge bases [49].

The general knowledge graph has a broad scope, a large scale, and a high degree of openness. However, due to its shallow research depth and relatively low accuracy, they cannot fully meet the requirements in some areas where there is a strong industry knowledge background.

Currently, there are few studies on KGCSS construction and application. However, there have been many results on domain knowledge graphs in other industries. GeoKG, which is represented with formal geographic knowledge, links discrete knowledge with the ability to query knowledge and solve geographic problems [50]. KG is used as a technical tool to link symptoms to diseases for efficient medical clinical decision making [51]. In the legal domain, KG is also used to manage case knowledge [52]. The maritime hazardous materials knowledge graph enables automatic determination of hazardous materials in isolation [53]. In addition, KG is used for knowledge management in education [54].

From the review above, KGCSS belongs to the domain of KG. Woo et al. [55] demonstrated the value and ability of KG to facilitate safe work practices. KG has been proven to improve security knowledge management because it helps to capture and prioritize knowledge through database searches and questionnaires, and to distribute important knowledge to specific working groups. Therefore, KGCSS should formally describe CSS and their interrelationships [56,57] and provide a systematical method of CSS knowledge management from the perspective of “classification” and “categorization” [58,59]. It is necessary and feasible to introduce KG into the field of CSS management in order to realize systematic knowledge management [60].

## 3. Materials and Methods

This study utilizes a top-down method to design the ontology of KGCSS. The top-down method refers to the design idea of defining the ontology of KG first and then adding entities to the knowledge base [61]. Ontologies are considered to be knowledge organization systems with elements that interact in a consistent conceptual structure [62]. Concept refers to the classification of a series of real objects. Entities are the most basic elements of KG and are the extrapolation of concepts which exist in the real world. Brainstorming is an effective way to utilize group wisdom and propose innovative ideas [63]. In this paper, we organized several seminars to discuss KGCSS construction. The participants included professionals from construction safety management authorities, standards management authorities and contractors. The research steps of KGCSS included knowledge structure analysis, conceptual layer design, entity layer construction and knowledge storage.

### 3.1. Knowledge Structure Analysis of CSS

CSS are divided into mandatory standards and recommended standards. Mandatory clauses are the most binding operational or regulatory requirements of mandatory standards. From the viewpoint of CSS release, CSS can be divided into national standards, industry standards, local standards, and corporate standards. In general, national standards have the largest scope of application, and enterprise standards have the smallest scope of application.

Knowledge structure analysis of CSS refers to the process of collecting standard documents, analyzing standard content, and dividing standard content levels. Knowledge structure reveals the components and levels of knowledge, which helps to understand CSS knowledge comprehensively and lay the foundation for the design of KGCSS. In order to analyze a CSS knowledge structure, we needed to obtain related materials from digital repositories.

**Figure 1:**
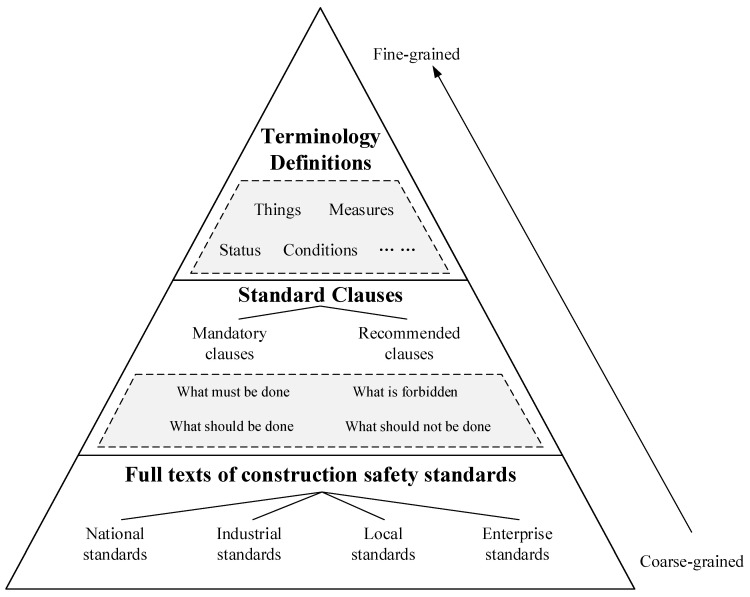
Structure analysis of construction safety standard knowledge.

Because official websites do not provide unified aggregation of CSSs, we aggregated related standards on websites, such as standard building library, national standard full text public system, and national standard information public service platform by searching for “construction management”, “construction technology”, and “construction safety”. There were 218 CSSs collected, including 65 national standards, 47 industry standards, 96 local standards, and 10 corporate standards. CSS knowledge can be divided into different granularity levels. Fine-grained knowledge is indivisible knowledge, and CSS fine-grained knowledge refers to the basic components of standard clauses. Coarse-grained knowledge is general and rough knowledge, and CSS coarse-grained knowledge refers to the full text of standards. Accordingly, the knowledge of CSS can be divided into the following three levels, and the knowledge structure is shown in Figure 1.

Terminology and definitions are basic components of standards and the basis of standard compilation. They include professional expression on objects and distinctive definitions of things, measures, status, and conditions in the construction industry.

Standard clauses are divided into mandatory and recommended clauses, including clauses that must be done, must not be done, recommended to be done, and should be done, etc. According to different objects, they can be divided into clauses for specialized construction matters, such as safety checks and firefighting, as well as specific targets like scaffolding and temporarily installed suspended access equipment.

The full texts of standards contain all the information of CSSs, including covers, tables of contents, prefaces, main contents, appendix, and explanation of clauses.

### 3.2. Construction of KGCSS

KGCSS is constructed in three steps: conceptual layer design, entity layer design, and knowledge storage.

#### 3.2.1. Conceptual Layer Design

**Figure 2:**
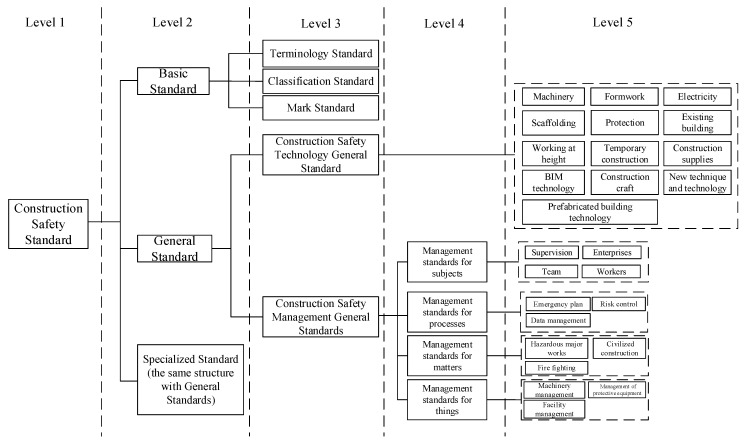
Conceptual layer framework of KGCSS.

The concept of KGCSS refers to the categories to which CSS belong through a rational division. The concept layer of KGCSS is a normative and structured representation of CSS knowledge. There are five levels of CSS concepts. The first level is CSS. The second level is divided into basic, general, and specialized standards according to their applicability. The third level is a subdivision according to different usage scenarios. The fourth level is a further division for management standards. The fifth level is a division for specific objects or matters. In each level, concepts are further divided into different categories to suit different types of construction tasks. Accordingly, the conceptual layer framework is shown in Figure 2.

From the second level of CSS concepts, CSS is divided into three categories.

Basic Standards serve as the foundation for the formulation of other CSSs in the system. They serve to unify concepts covered by other standards and to minimize disputes in the compilation and application process caused by different interpretations of industrial terminology in the field of construction. Terminology Standards provide a unified language in the compilation process. Classification Standards put forward a criterion for reasonable division. Mark Standards specify the setting and maintenance of safety signs on the construction site. These three are defined as Basic Standards.

General Standards are unified standards that aim to regulate specific construction stages (such as transformation) and matters (such as electricity consumption), including general standards for construction safety technology and management. Construction Safety Technology General Standards make specific and specialized requirements for the operation of tools and the implementation of critical construction methods, including machinery, scaffolding, formwork, electricity, temporary construction, working at height, and construction supplies. Among the Construction Safety Management General Standards are management standards for subjects that provide guidance to safety management organizations, such as construction enterprises and supervision departments. Management standards for processes make provisions to regulate construction processes, including risk control, security check and other processes. Management standards for matters aim to guarantee important construction safety matters, such as hazardous major works. Management standards for things ensure the safe utilization of apparatuses in construction.

Compared to General Standards, Specialized Standards have a comparable structure to General Standards but relate to more specific management and technical objects.

Correspondingly, relationships between concepts in the KGCSS are organized into eight categories which are shown in Table 1.

#### 3.2.2. Entity Layer Design

The entities of KGCSS are current CSS. KGCSS entity layer is a collection of clearly classified CSS corresponding to KGCSS concepts. The steps of KGCSS entity layer construction include: entity identification, attribute extraction, and entity extraction.

**Figure 3:**
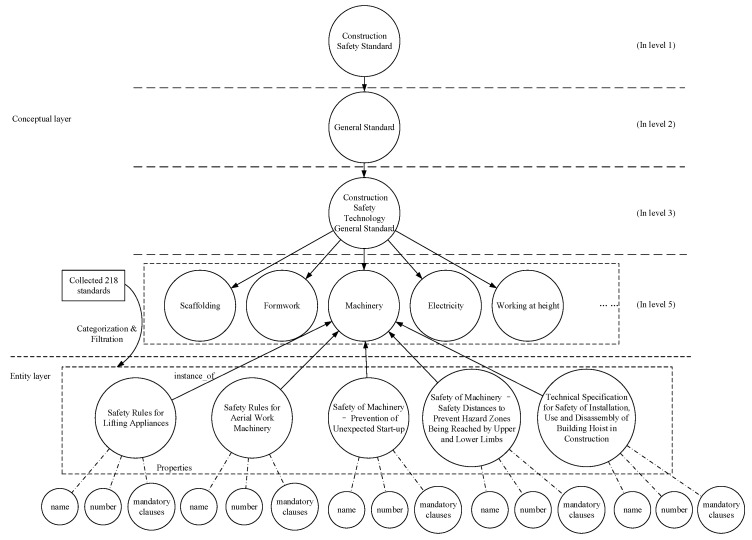
Construction logic of the entity layer of KGCSS.

The 218 current standards collected in Section 3.1 are pending entities to be identified and categorized in accordance with CSS concepts. Furthermore, the names, numbers and mandatory clauses of current standards are extracted as properties of entities. Based on the structure of the conceptual layer, the collected 218 CSS were sorted and filtered to form an entity layer, which finally contains 147 standard entities. The relationships from the entity to the corresponding concept are designed with the name “is an instance of”. The construction logic of the entity layer is shown in Figure 3.

Entity extraction is the process of matching, classifying, and filtering entities with concepts one by one. It is a unique step in the establishment of KGCSS. For instance, the name “Safety Rules for Lifting Appliances” contains two main parts: “working at height” and “appliances”, which correspond to the concepts of “working at height” and “machinery” respectively (in Figure 2). Therefore, it is necessary to identify a conceptual classification for the standard according to its main content. The “Safety Rules for Lifting Appliances” applies to aerial work platforms and aerial work vehicles. It aims to guide the design, manufacture, use, maintenance, and management of aerial work machinery products. It also contains several mechanical terms, such as leveling mechanisms, wire ropes and hydraulic systems. In addition, the requirements for users are to maintain normal operation of the machinery, rather than guiding workers on how to work at height. Therefore, “Safety Rules for Lifting Appliances” should be classified as general standards for machinery technology. The materialization process is shown in Figure 3. In addition, standards will be removed from the entities if they do not meet any concept, as in the example of the general safety technology standard; the results of extracting entities are shown in Table 2.

#### 3.2.3. Knowledge Storage

Knowledge storage refers to importing knowledge into a database to serve upper-layer applications, such as knowledge presentation, knowledge reasoning and intelligent Q&A. The knowledge of CSSs is stored in the Neo4j database in this research. Neo4j supports operational functions, including add, delete, and modify, making it convenient to adjust the design of KGCSS [64]. Organized CSS knowledge is stored in a Neo4j database via py2neo module in Pycharm. The knowledge graph contains 177 nodes, 263 relations and 11 properties. The result of knowledge storage is shown in Table 3.

## 4. Results

Based on the KGCSS constructed in Section 3, this subsection implements KGCSS in practice, including automatic reasoning of CSS planning knowledge retrieval based on natural language processing.

### 4.1. Automatic Reasoning of CSS Planning

Standard planning can be achieved from the current CSS system via knowledge reasoning. CSS planning involves a list of standard work plans that serve as a roadmap for CSS development and revision. Planning standards are derived from “Missing Standards”, “Standards to be Integrated” and “Standards That Require Complementary Knowledge”. First, standards that are absent from the list of basic and general standards are “Missing Standards”, such as “Construction Safety Terminology Standard”. In addition, under the same concept, standards with similar themes should be regarded as “Standards to be Integrated” [19]. For instance, “Temporarily Installed Suspended Access Equipment”, “Suspended Powered Platforms for Work at Height-safety Regulation”, and “Technical Specification for Installation Dismantlement and Operation of Temporarily Installed Suspended Access Equipment” are all intended to regulate the implementation of temporarily installed suspended access equipment, and as such they should be integrated and unified as “Safety Technical Regulations for Temporarily Installed Suspended Access Equipment”. Additionally, “Standards That Require Complementary Knowledge” refers to standards that are present in General Standards but not in Specialized Standards, such as “Safety Code for Tower Cranes”.

Moreover, planning standards are divided into four levels of priorities to indicate the importance and compilation urgency of each standard. Priorities are determined based on the planning standards’ attributes. Missing basic or general standards are the first priority. “Standards to be Integrated” are determined as the second priority. The third priority is set for “Standards That Require Complementary Knowledge”. Missing specialized standards are the fourth priority. Results of the CSS plan is shown in Table 4.

### 4.2. Knowledge Retrieval Based on Natural Language Processing

Natural language is the language used in daily human communication. The CSS knowledge Q&A function relies on domain entities and conducts reasoning based on KGCSS to answer users’ questions in depth [53]. Knowledge Q&A is better at addressing knowledge-based questions and is more direct and intuitive in providing replies than template-based chatbots. This subsection implements an automatic function of Q&A in the KGCSS via question classification, rule-based matching, query statement generation and answer template invocation [32].

First, types of questions are defined and classified. Common problems of standard users have been identified according to the results of expert seminars. Questions are divided into five categories, including: “latest version of the standard”, “mandatory clauses of the standard”, “standards involved in a certain field”, “mandatory clauses involved in a certain field”, and “standard planning in a certain field”. 

**Figure 4:**
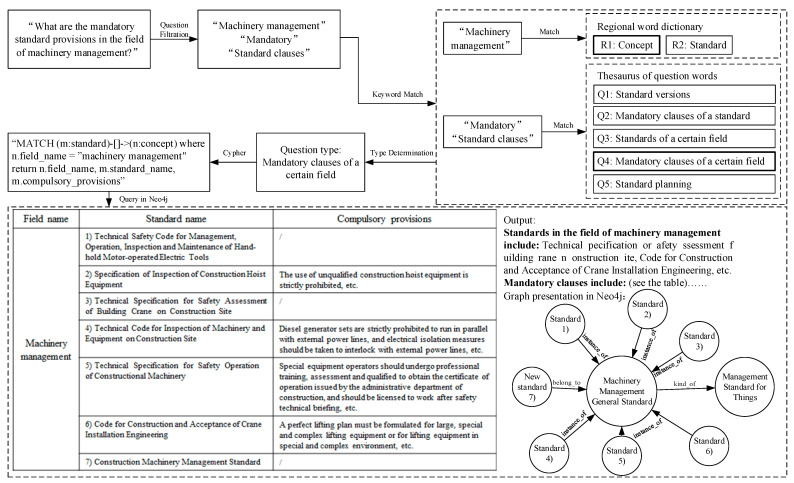
Q&A function of KGCSS.

Second, matching rules are defined for each question. Matching rules specify what conditions need to be met for a question sentence to be classified as a type of question we designed. The matching rules include a feature word lexicon and a question word bank. Feature words include concepts and entities of KGCSS. Question words refer to the objects of question sentences. When both feature words and question words contained in a question sentence match the rules of a question type, the sentence is classified as that type of question. For example, when the sentence “What are the mandatory standard clauses in the field of machinery management?” is typed in, the question type is obtained by matching as “mandatory clauses involved in a certain field”, as shown in Figure 4. 

Third, query statements are designed for the five types of questions in order to activate the knowledge in KGCSS and answer templates. In other words, after the question is classified, the query statement will invoke its feature terms, search information in KGCSS and present the answer. The process and outcomes of the question “What are the mandatory standard clauses in the field of machinery management?” are shown in Figure 4.

## 5. Discussion

### 5.1. Innovation

CSS contains rich domain knowledge, which provides important materials for ensuring construction safety. The KGCSS constructed in this paper realizes CSS knowledge management, fills the research gap, solves the problems existing in the application of CSS, and has strong practical significance. The main innovations of this paper are as follows.

(1) KGCSS is a universal and operational result for CSS management. It realizes systematical integration of CSS knowledge and better standard management.
(2) The standard planning is obtained based on KGCSS reasoning, which is the most practical result of this paper. CSS planning is oriented to users’ needs and standard content, clarifying the future work tasks of CSS, and making CSS development and revision more in line with market needs.
(3) Q&A function of KGCSS is designed. Compared with existing standard query websites, the CSS Q&A function realizes a knowledge search with the precision of clauses. Systematically integrated CSS knowledge is conducive to guiding workers’ behavior and supporting users’ decision making.

However, the exploration of CSS knowledge management is far from complete. Although KGCSS realizes deeper standard knowledge management, what KGCSS currently achieves is merely to communicate knowledge to users rather than assisting them how to make use of it, i.e., there is no guarantee of knowledge dissemination. In previous studies, technical requirements were translated into actionable instructions for workers through construction practice surveys [29]. However, the issue of how to achieve efficient dissemination of all standards in KGCSS remains to be addressed. Additionally, in other domains, machine learning methods and KG are combined for knowledge management, enabling more powerful applications, such as optimizing methods of knowledge representation of GeoKG [50] and distinguishing reliable relation paths among large amounts of meaningless relation paths [65]. Nevertheless, the knowledge in KGCSS is only accurate to the “clause” level, and query results of mandatory clauses are output in the form of paragraphs. The exploitability of KGCSS-based applications depends on the availability of a more formalized CSS knowledge structure, which also needs to be investigated in depth in the future.

### 5.2. Research Prospects

As a knowledge management tool, knowledge graph has broad research prospects. Based on this paper, future research of KGCSS can be focused on the following aspects.

Knowledge representation: the knowledge representation of CSSs solely reaches the accuracy of “clauses”, and mandatory clauses are only output as a whole paragraph. Thus, an event extraction algorithm can be introduced to create a structured representation of standard clause knowledge and to aid in more advanced knowledge reasoning.

Platform construction: the KGCSS can be used to establish an information platform accessible to the government and construction enterprises. With the accumulation of knowledge on the platform via standard users uploading their safety production experiences, experience feedback will serve as industrial knowledge to enrich current construction safety knowledge, as well as function as references to solve problems on various projects [66].

## 6. Conclusions

Given that much attention has been paid to CSS management, the purpose of this paper is to improve CSS application and management. Knowledge characteristics of CSS make them suitable as objects for knowledge management [21]. In view of this, this paper employs KG techniques for systematic management of CSS. Firstly, a three-level CSS structure is obtained by analyzing the knowledge of 218 CSSs. Secondly, based on the expert opinions, a conceptual layer design is carried out, and a five-level CSS concept layer is obtained. The 218 standards are reasonably correlated to the conceptual layer to form an entity layer. Finally, knowledge storage is used to construct KGCSS, which contains 147 entities and 177 nodes.

This paper achieves significant improvements in methodological applications and results of CSS management. First, this paper proposes a novel standard planning based on KGCSS.A CSS revision plan is deduced, including twenty-one “Missing Standards”, seven “Standards to be Integrated” and eight “Standards That Require Complementary Knowledge”. The standards plan is a valuable tool for meeting the most urgent needs of CSS usage, such as government’s supervision and contractors’ production. The plan provides recommendations for standard revisions and improves the completeness of CSS knowledge. In addition, a CSS query function has also been devised and implemented. Unlike the general standard query platform, it offers CSS knowledge at a granularity of clauses rather than the entire standard documents. However, the existing CSS knowledge presentation is less structured, which should be further addressed in future studies.

Nevertheless, there are still some limitations in our research. First, the KGCSS does not collect all of local standards in China due to resource limitation. A KGCSS of rich local standards applicable to different regions can be improved in the future. Moreover, this study solely considers standards as KGCSS entities. More detailed knowledge representation, such as regarding terminology as entities and developing their connections, should be explored in future research.

## Figures and Tables

**Table 1:** Concept relationships of construction safety standard knowledge graph.

Relation Types	Explanation
Basis of compilation	It is the relation between Basic Standards and Construction Safety Standards, denoting that standard compilation should comply with terminology and definitions in Basic Standards.
All categories applicable	It is the relation between General Standards and Construction Safety Standards, denoting applicable standards can be found in General Standards for all types of construction projects.
Specific objects applicable	It is the relation between Specialized Standards and Construction Safety Standards.
Kind of	It is the classification relation between concepts.
Classified by subjects	It is the relation between management standards for subjects and construction safety management standards.
Classified by processes	It is the relation between management standards for processes and construction safety management standards.
Classified by matters	It is the relation between management standards for matters and construction safety management standards.
Classified by things	It is the relation between management standards for things and construction safety management standards.

**Table 2:** Entities of construction safety standard knowledge graph (part).

Concepts	Entities (Current Standards)
Machinery Technology General Standard	Safety Rules for Lifting Appliances Technical Specification for Safety of Installation, Use and Disassembly of Building Hoist in Construction Safety of Machinery: Prevention of Unexpected Start-up Safety of Machinery: Safety Distances to Prevent Hazard Zones being Reached by Upper and Lower Limbs Safety Rules for Aerial Work Machinery
Mold Frame Technology General Standard	Technical Code for Safety of Forms in Construction Technical Code for Temporary Support Structures in Construction
Scaffolding Technology General Standard	Unified Standard for Safety of Scaffold in Construction
Electricity Use Technology General Standard	General Guide for Safety of Electric User Code for Safety of Power Supply and Consumption for Construction Site Technical Code for Safety of Temporary Electrification on Construction Site
Work-at-height Technology General Standard	Technical Code for Safety of Working at Height of Building Construction
……	……

**Table 3:** Aggregation of construction safety standard knowledge storage results.

Knowledge Types		Quantity	Examples
Concepts	Level 1	1	Construction Safety Standard
	Level 2	3	Basic Standard, General Standard, Specialized Standard
	Level 3	4	Construction Safety Management General Standard, Construction Safety Technology General Standard, Construction Safety Management Specialized Standard, Construction Safety Technology Specialized Standard
	Level 4	8	Construction Safety Management General Standard for Subjects, Construction Safety Management General Standard for Processes, etc.
	Level 5	51	Enterprise Safety Management General Standard, Safety Plan General Standard, Safety Management General Standard for Dangerous and Large Projects, etc.
Entities	Current standards	111	Technical Code for Safety of Lifting in Construction, Technical Code for Fire Safety of Construction Site, Safety Rules for Lifting Appliances—Part1: General, etc.
Properties	Standard numbers	111	GB2893-2020, GB2894-2018, JGJ59-2011, etc.
	Mandatory clauses	58	What should be done, what is forbidden, etc.
	……	……	……
Relations	Relations between concepts	8	See Table 1
	Relations between entities	1	Instance of

**Table 4:** Construction safety standard planning.

Priority	Quantity	Names of Planning Standards
1st	20	Construction Safety Terminology Standard, Construction Material Classification Standard, Risk Level Classification Standard, Construction Project Safety Supervision Standard, Construction Project Team Safety Management Standard, etc.
2nd	7	Construction Machinery Safety Technology Unified Standard, Construction Site Temporary Electricity Safety Technology Standard, Construction Mold Frame Unified Safety Technology Standard, Construction Scaffolding Unified Safety Technology Standard, etc.
3rd	8	Fixed Scaffolding Safety Technical Standard, Mobile Scaffolding Safety Technical Standard, Tower Crane Safety Technical Standard, Prefabricated Building Safety Technical Standard, etc.
4th	1	New Technology, New Techniques, New Materials, New Equipment and Safety Contents involved

## Data Availability

Data sharing not applicable.
